# Comparative Brain and Serum Exosome Expression of Biomarkers in an Experimental Model of Alzheimer-Type Neurodegeneration: Potential Relevance to Liquid Biopsy Diagnostics

**DOI:** 10.3390/ijms26094190

**Published:** 2025-04-28

**Authors:** Suzanne M. de la Monte, Yiwen Yang, Anjali Prabhu, Ming Tong

**Affiliations:** 1 Departments of Medicine, Pathology and Laboratory Medicine, Neurology, and Neurosurgery, Rhode Island Hospital, Women & Infants Hospital, Brown University Health, The Warren Alpert Medical School of Brown University, Providence, RI 02903, USA; 2 Graduate Program in Biotechnology, Brown University, Providence, RI 02912, USA; yiwenyang@umass.edu (Y.Y.); anjali_prabhu@alumni.brown.edu (A.P.); 3 Department of Medicine, Rhode Island Hospital, Brown University Health, The Warren Alpert Medical School of Brown University, Providence, RI 02903, USA; mtong216@gmail.com

**Keywords:** Alzheimer’s disease, liquid biopsy, exosomes, white matter, streptozotocin, rat model, oxidative stress, amyloid, phospho-tau, oligodendrocytes

## Abstract

The development of more effective disease-modifying treatments for Alzheimer’s disease (AD) is compromised by the lack of streamlined measures to detect and monitor the full spectrum of neurodegeneration, including white matter pathology, which begins early. This study utilized an established intracerebral streptozotocin (STZ) model of AD to examine the potential utility of a non-invasive serum extracellular vesicle (SEV)-based liquid biopsy approach for detecting a broad range of molecular pathologies related to neurodegeneration. The design enabled comparative analysis of immunoreactivity in frontal lobe tissue (FLTX), frontal lobe-derived EVs (FLEVs), and SEVs. Long Evans rats were administered i.c. STZ or saline (control) on postnatal day 3 (P3). Morris Water Maze testing was performed from P24 to P27. On P31–32, the rats were sacrificed to harvest FLTX and serum for EV characterization. STZ caused brain atrophy, with deficits in spatial learning and memory. STZ significantly impacted FLEV and SEV nanoparticle abundance and size distributions and concordantly increased AD (Tau, pTau, and Aβ) and oxidative stress (ubiquitin, 4-HNE) biomarkers, as well as immunoreactivity to immature oligodendrocyte (PLP), non-myelinating glial (PDGFRA, GALC) proteins, MAG, nestin, and GFAP in FLTX and FLEV. The SEVs also exhibited concordant STZ-related effects, but they were limited to increased levels of 4-HNE, PLP, PDGFRA, GALC, MAG, and GFAP. The findings suggest that non-invasive EV-based liquid biopsy approaches could potentially be used to detect and monitor some aspects of AD-type neurodegeneration. Targeting brain-specific EVs in serum will likely increase the sensitivity of this promising non-invasive approach for diagnostic and clinical management.

## 1. Introduction

Alzheimer’s disease (AD) is a complex, multifaceted disease that remains difficult to diagnose, particularly in its early stages. Effective therapeutic interventions have been slow to emerge due to the time-consuming nature of clinical assessments and the need to better understand the full spectrum of pathologies contributing to cognitive decline, including white matter degeneration, microvascular disease, blood–brain barrier dysfunction, and perturbations in energy metabolism, mitochondrial function, neuroinflammatory homeostasis, and antioxidant neuroprotection [1,2]. The decades-long downward spiral of neurodegeneration [3] provides opportunities to intervene by addressing different facets and stages of disease via the implementation of sensitive screening tools that exceed the resolution, speed, and accessibility and undercut the costs of neuroimaging and neuropsychological tests. The current emphasis on AD diagnostics is centered around the progressively changing core of biomarkers linked to the ATN (Amyloid, Tau, neurodegeneration) framework, which acknowledges AD’s complexity and utilizes resources to generate relevant data via the analysis of body fluids, neuroimaging, and the integration of common co-pathologies [4,5]. Liquid biopsy, exosome/extracellular vesicle (EV)-based approaches offer excellent prospects to enhance the detection and monitoring of a broad spectrum of AD-related brain pathology. Proof-of-concept data have already emerged demonstrating the utility of liquid biopsy-based non-invasive diagnostic approaches to malignancies [6,7,8,9,10,11,12], neurodegenerative diseases [13,14,15,16,17,18,19], and neurodevelopmental disorders [20,21].

EVs are lipid bilayer membrane-bound particles released by cells into extracellular spaces. EVs constitute a heterogeneous group of particles, subdivided into three main classes, exosomes (20 nm–150 nm), microvesicles (150 nm–1000 nm), and apoptotic bodies (100 nm–5000 nm), although evidence has emerged that much larger non-vesicular extracellular nanoparticles also contribute to inter-cellular communication [22]. EVs express tetraspanins and share the capacity to carry and transport molecular cargo composed of proteins, nucleic acids, and lipids, but, morphologically, they differ in size and function [22]. Exosomes are shed from every cell type and are readily distributed in biological fluids, including serum, tears, saliva, urine, and cerebrospinal fluid [15,22,23,24]. In addition, they retain cellular- and tissue-specific membrane markers that could be used to identify organ/tissue disease sources [25,26,27]. Liquid biopsy strategies are highly desirable for diagnosing and monitoring neoplastic, neurodegenerative, inflammatory, and ischemic diseases [28,29,30,31,32,33,34,35,36,37] due to their rich arrays of protein and nucleic acid cargo [38]. The ability to isolate EVs from various body fluids renders them suitable for non-invasive or minimally invasive diagnostic approaches [39]. For example, regarding neurodegenerative diseases, researchers have shown that disease-relevant exosomes isolated from serum and CSF [40] carry cargo reflecting neuronal proteins abnormally expressed in AD, frontotemporal lobar degeneration, motor neuron disease, Parkinson’s disease, or Creutzfeldt–Jakob, although the specificity of the findings has been challenging [41].

White matter (WM) pathology, including myelin loss and atrophy, is a significant feature of AD neurodegeneration and is likely mediated by the targeting of oligodendrocytes [2,42,43,44], which synthesize and maintain myelin needed to ensure efficient speeds of neurotransmission [45]. In addition, astrocyte dysfunction impairs blood–brain barrier and synaptic integrities [46], and microglia activation drives neuroinflammatory responses that exacerbate damage mediated by oligodendrocyte degeneration and astrocyte activation. Our working hypothesis is that, due to their widespread presence in WM [47], AD-associated alterations in myelin oligodendrocyte glycoprotein expression, astrocyte activation, axonal breakdown, and neuroinflammation with oxidative stress and lipid peroxidation would be detectable in exosomes isolated from brain tissue, CSF, and plasma. Such abnormalities have been well described in AD as well as other forms of neurodegeneration like alcohol-related brain disease [48].

Unlike the currently available CSF- and serum-based assays for measuring Amyloid beta (Aβ), phosphorylated Tau (pTau), neurofilament light chain for neuronal loss, or pro-inflammatory cytokines/chemokines as diagnostic aids for AD and other neurodegenerative diseases [17,49,50,51,52,53,54], there are no such tools available for detecting and monitoring WM degeneration in AD. Previous studies of an experimental intracerebral (i.c.) streptozotocin (STZ) model demonstrated AD-type neurodegeneration with deficits in spatial learning and memory [55,56,57] and WM molecular, biochemical, and signal transduction abnormalities corresponding to impairments in myelin/oligodendrocyte function [56,57]. For the present study, we evaluated the potential utility of a liquid biopsy approach for detecting multiple facets of AD-type neurodegeneration, i.e., myelin/oligodendrocyte, astrocyte, oxidative stress, and standard pTau and Aβ biomarkers in EVs isolated from frontal lobe tissue (FLEV) and serum of i.c. STZ models. In addition, parallel studies with FLTX homogenates were used to compare how abnormalities in the brain are sorted into EVs.

## 2. Results

**Figure 1:**
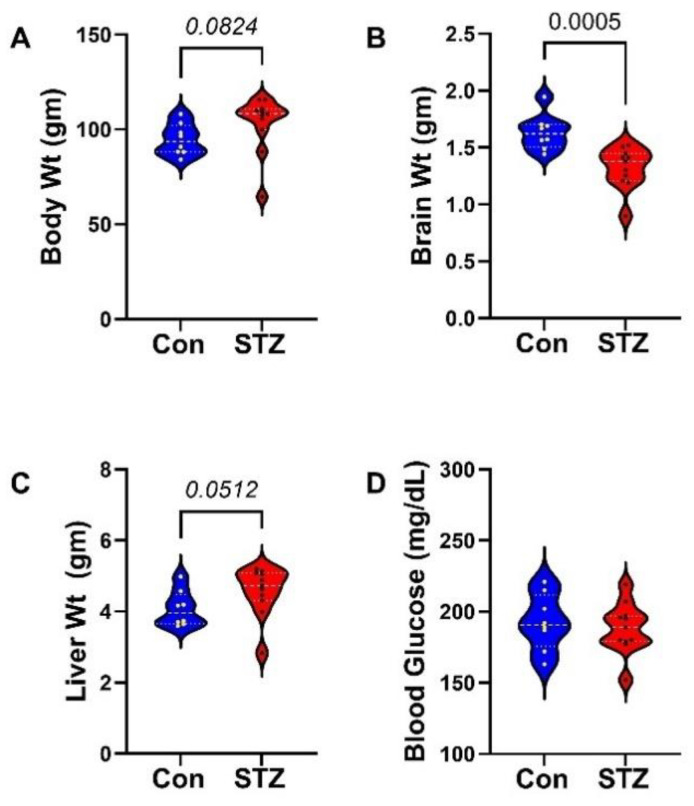
Effects of i.c. STZ on body, brain, and liver weights and blood glucose levels. Long Evans male and female rats were treated by intracerebral injection of STZ (0.8 mg/kg) on postnatal day 3 (P3). P30 was the experimental endpoint. Violin plots depict the STZ effects on terminal mean (±S.D.) of (**A**) body weights, (**B**) brain weights, (**C**) liver weights, and (**D**) blood glucose. Intergroup comparisons were made by two-tailed *t*-tests. Significant differences correspond to *p* < 0.05. Statistical trend-wise differences (italicized) reflect 0.05 < *p* < 0.10.

**Figure 2:**
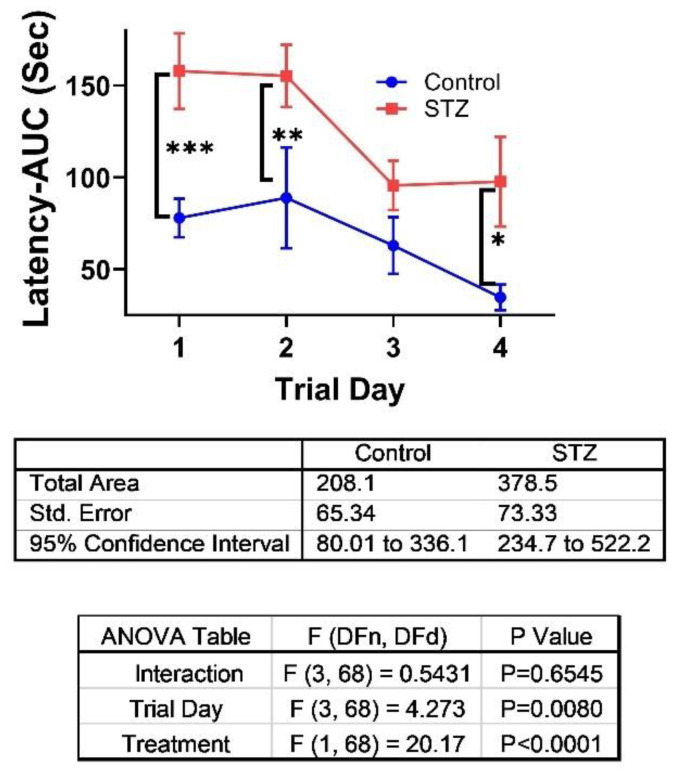
Effects of i.c. STZ on spatial learning and memory Morris Water Maze (MWM) Test. MWM testing was performed on four consecutive days from postnatal day (P) 25 through P28. The latencies required to reach and land on the platforms were tracked and scored with Ethovision 8.5 (Noldus, Leesburg, VA, USA) software. Area-under-curve (AUC) calculations over the 3 daily trials were used for inter-group comparisons. The graph depicts the linear trajectories of performance over the 4 Trial Days. Data points reflect the mean + S.D. of the latencies. The AUC total mean ± S.E.M. and the two-way ANOVA test results are tabulated below the graph. Significant (*p* < 0.05) post hoc intergroup differences. * *p* < 0.05; ** *p* < 0.01; *** *p* < 0.001.

STZ Effects: The i.c. STZ treatment trend-wise increased the mean body (Figure 1A) and liver (Figure 1C) weights but significantly reduced brain weight (*p* = 0.0005; Figure 1B) without affecting mean blood glucose (Figure 1D). The absence of hyperglycemia confirms that the i.c. STZ treatment did not cause diabetes mellitus. MWM testing revealed significantly longer latencies in the STZ group relative to control on Trial Days 1, 2, and 4 (Figure 2). Correspondingly, area-under-the-curve analysis was significantly higher in the STZ group (Figure 2). The two-way ANOVA test demonstrated significant effects of treatment (*p* < 0.0001) and Trial Day (*p* = 0.008) (Figure 2). However, it is noteworthy that performance improvements over time occurred in both groups between Trial Days 2 and 4.

**Figure 3:**
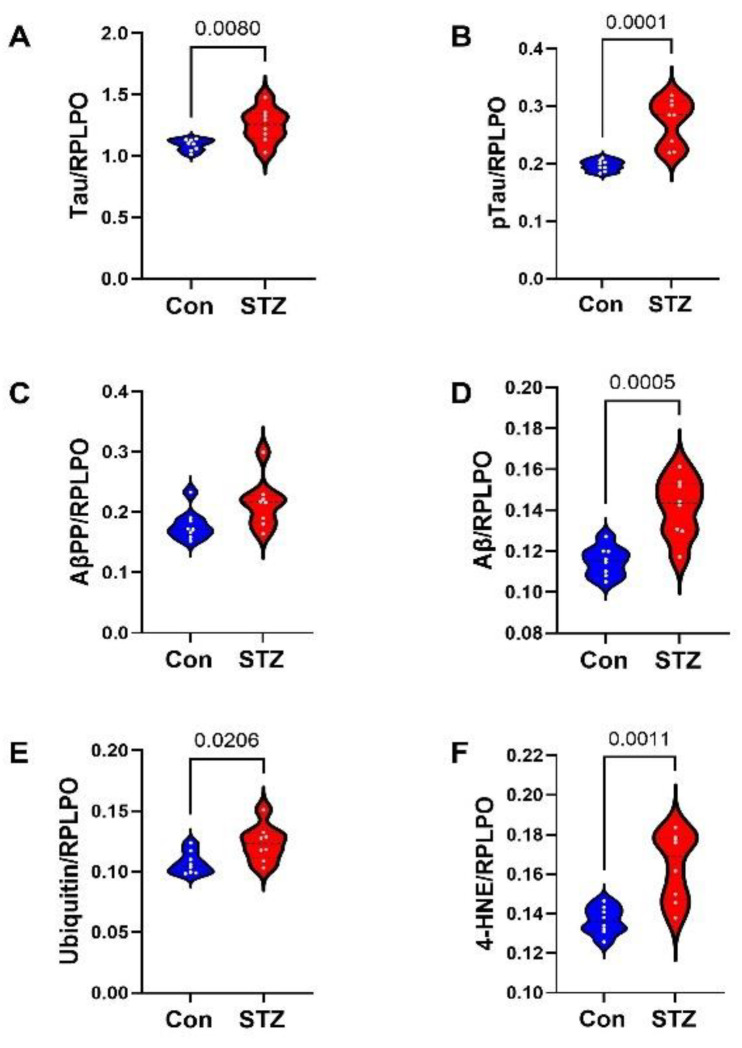
FLTX AD and oxidative stress biomarkers: Violin plots compared FLTX levels of (**A**) Tau, (**B**) pTau, (**C**) AβPP, and (**D**) Aβ1-42, (**E**) ubiquitin, and (**F**) 4-HNE immunoreactivities in control (Con) and i.c.-STZ (STZ) treated frontal lobe tissue from Long Evans P30 rats (n = 8/group). Immunoreactivity was measured by duplex ELISA with results normalized to RPLPO. Intergroup comparisons were made by two-tailed *t*-tests with Bonferroni correction for repeated measures. Significant differences correspond to *p* < 0.05.

**Figure 4:**
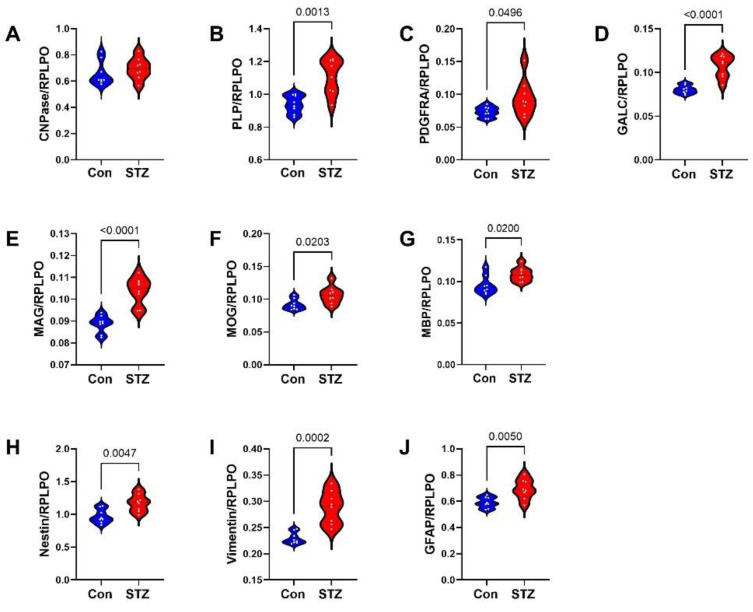
FLTX glial protein expression: FTLX samples from control (Con) and i.c. STZ (STZ) treated P30 Long Evans rats were used to measure (**A**) CNPase, (**B**) PLP, (**C**) PDGFRA, (**D**) GALC, (**E**) MAG, (**F**) MOG, (**G**) MBP, (**H**) Nestin, (**I**) vimentin, and (**J**) GFAP by duplex ELISA with results normalized to RPLPO. Assays were performed in triplicate with 8 samples/group. Intergroup comparisons were made by two-tailed *t*-tests with Bonferroni correction for repeated measures. Significant differences correspond to *p* < 0.05.

Frontal Lobe Tissue (FLTX) Studies: FLTX ELISA studies demonstrated significantly higher levels of Tau (*p* = 0.008; Figure 3A), pTau (*p* = 0.0001; Figure 3B), Aβ (*p* = 0.0005; Figure 3D), ubiquitin (*p* = 0.02; Figure 3E), and 4-HNE (*p* = 0.001; Figure 3F) in STZ relative to control and no significant effects of i.c.-STZ on AβPP (Figure 3C). The glial protein studies included markers of immature, non-myelinating oligodendrocytes (CNPase, PLP, PDGFRA, GALC) (Figure 4A–D), myelinating oligodendrocytes (MAG, MOG, MBP) (Figure 4E–G), and astrocytes (Nestin, vimentin, GFAP) (Figure 4H–J). All markers, except CNPase, were significantly elevated in the STZ relative to control FLTX samples.

**Figure 5:**
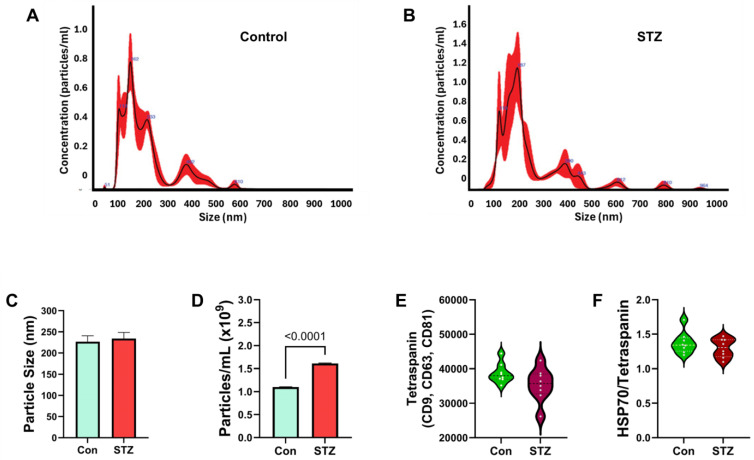
FLEV exosome characterization: FLEVs were analyzed for nanoparticle size distribution and abundance using Nanotracker Analysis (NTA). Exosomes were isolated from frontal lobe tissue on P30 (experimental endpoint). Representative NTA distribution profiles in (**A**) control and (**B**) STZ samples are shown. The NTA software (v3.44) calculated the mean (±S.D.) (**C**) nanoparticle diameters (nm) and (**D**) concentration (particles/mL). Violin plots depict (**E**) Tetraspanin and (**F**) HSP70/tetraspanin immunoreactivities measured by ELISA in 50 ng FLEV exosome protein samples. Assays were performed in triplicate with 8 samples/group. Intergroup comparisons were made by two-tailed *t*-tests with Bonferroni correction for repeated measures. Significant differences correspond to *p* < 0.05.

Frontal Lobe EV Studies: Nanotracker analysis (NTA) of FLEVs revealed differences in the particle size profiles between control and STZ samples associated with broader distributions above 250 nm in the STZ group (Figure 5A,B). However, the mean nanoparticle diameters were similar for the two groups (Figure 5C), but the concentrations were significantly elevated by STZ treatment (Figure 5D). ELISA studies demonstrated similar levels of tetraspanin (Figure 5E) and HSP70/tetraspanin (Figure 5F) immunoreactivity in 50 ng FLEV protein from control and STZ samples. Correspondingly, Western blot analysis detected tetraspanin immunoreactivity in EVs isolated from both FL and serum samples of control and i.c. STZ-treated rats (Supplementary Figure S1).

**Figure 6:**
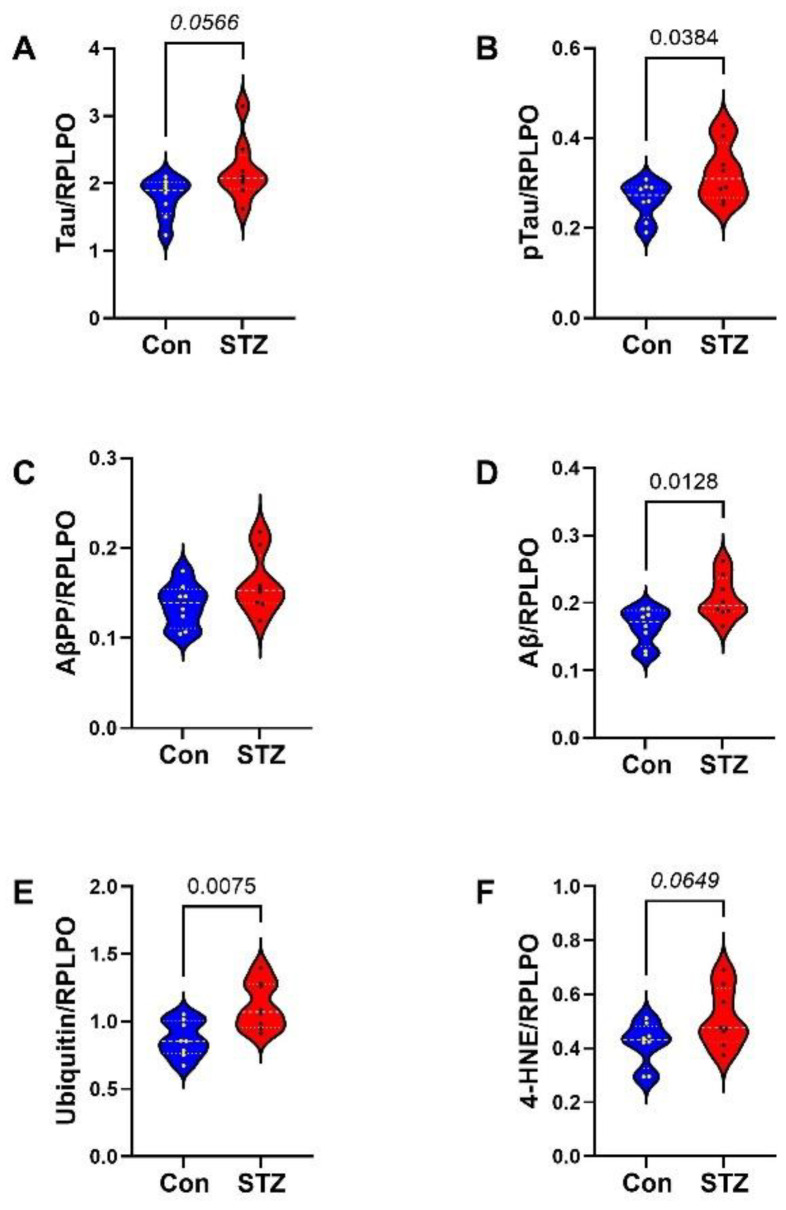
FLEV AD and oxidative stress biomarkers: Violin plots compared FLTX levels of (**A**) Tau, (**B**) pTau, (**C**) AβPP, and (**D**) Aβ1-42, (**E**) ubiquitin, and (**F**) 4-HNE immunoreactivities in control (Con) and i.c.-STZ (STZ) FLEVs isolated from Long Evans P30 rat brains (n = 8/group). Immunoreactivity was measured by duplex ELISA with results normalized to RPLPO. Intergroup comparisons were made by two-tailed *t*-tests with Bonferroni correction for repeated measures. Significant differences correspond to *p* < 0.05. Statistical trend-wise differences (italicized) reflect 0.05 < *p* < 0.10.

**Figure 7:**
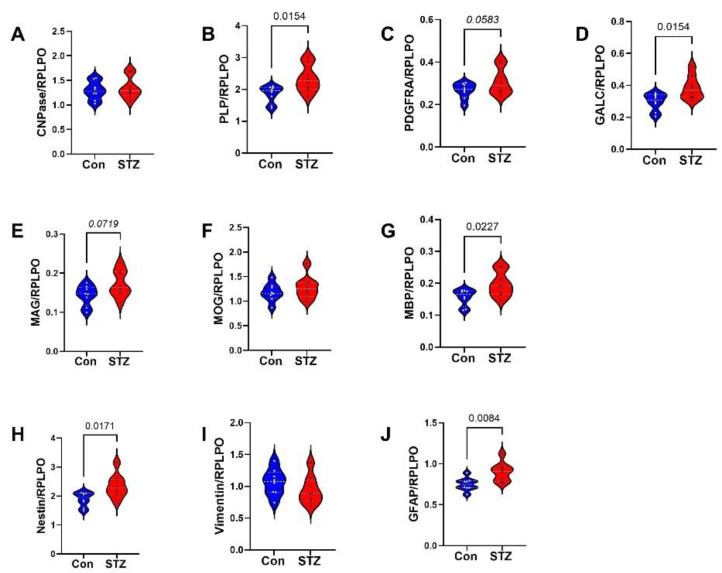
FLEV glial protein expression: FTEV samples from control (Con) and i.c. STZ (STZ) treated P30 Long Evans rats were used to measure (**A**) CNPase, (**B**) PLP, (**C**) PDGFRA, (**D**) GALC, (**E**) MAG, (**F**) MOG, (**G**) MBP, (**H**) Nestin, (**I**) vimentin, and (**J**) GFAP by duplex ELISA with results normalized to RPLPO. Assays were performed in triplicate with 8 samples/group. Intergroup comparisons were made by two-tailed *t*-tests with Bonferroni correction for repeated measures. Significant differences correspond to *p* < 0.05. Statistical trend-wise differences (italicized) reflect 0.05 < *p* < 0.10.

Duplex ELISAs measured AD biomarkers and glial immunoreactivities in FLEVs, with the results normalized to RPLPO for correlation with the FLTX data. Two-tailed *t*-tests with Bonferroni correction for multiple comparisons revealed significantly higher levels of pTau (Figure 6B), Aβ (Figure 6D), and ubiquitin (Figure 6E) and statistical trend-wise increases in Tau (Figure 6A) and 4-HNE (Figure 6F) in the STZ group but no significant or trend-wise effects of STZ on AβPP (Figure 6C). Regarding the glial proteins, STZ significantly increased the FLEV levels of PLP (Figure 7B), GALC (Figure 7D), MBP (Figure 7G), Nestin (Figure 7H), and GFAP (Figure 7J) and increased statistically trend-wise for PDGFRA (Figure 7C) and MAG (Figure 7E). In contrast, there were no significant or trend-wise effects of STZ on CNPase (Figure 7A), MOG (Figure 7F), or vimentin (Figure 7I) in FLEVs.

**Figure 8:**
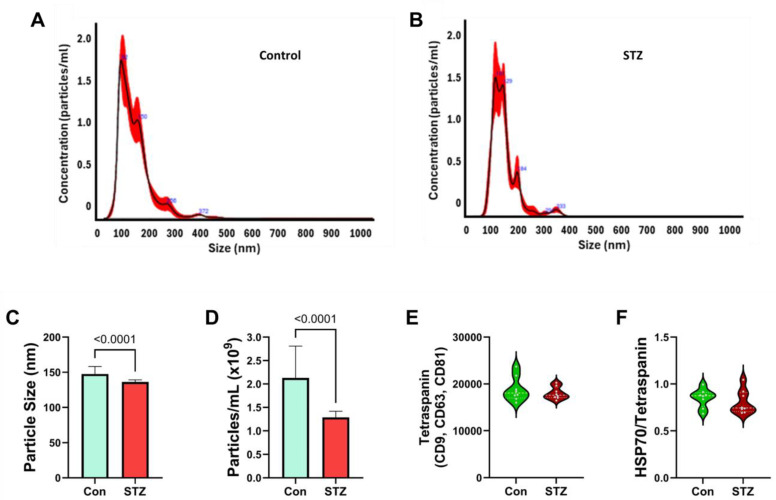
SEV exosome characterization: SEVs were analyzed for nanoparticle size distribution and abundance using Nanotracker Analysis (NTA). Exosomes were isolated from serum of P30 rats (experimental endpoint). Representative NTA distribution profiles in (**A**) control and (**B**) STZ samples are shown. The NTA software (v3.44) calculated the mean (±S.D.) (**C**) nanoparticle diameters (nm) and (**D**) concentration (particles/mL). Violin plots depict (**E**) Tetraspanin and (**F**) HSP70/tetraspanin immunoreactivities measured by ELISA in 50 ng SEV exosome protein samples. Assays were performed in triplicate with 8 samples/group. Intergroup comparisons were made by two-tailed *t*-tests with Bonferroni correction for repeated measures. Significant differences correspond to *p* < 0.05.

Serum EV (SEV) Analysis: The NTA of SEVs revealed a narrower size distribution profile in the STZ compared with control samples (Figure 8A,B). Correspondingly, STZ treatment resulted in a significantly smaller mean diameter nanoparticle (SEV) size relative to control (Figure 8C). In addition, the mean nanoparticle concentration was significantly lower in the STZ samples (Figure 8D). As observed for the FLEVs, the levels of tetraspanin (Figure 8E) and HSP70/tetraspanin (Figure 8F) immunoreactivity in 50 ng SEV protein were similar for the STZ and control groups.

**Figure 9:**
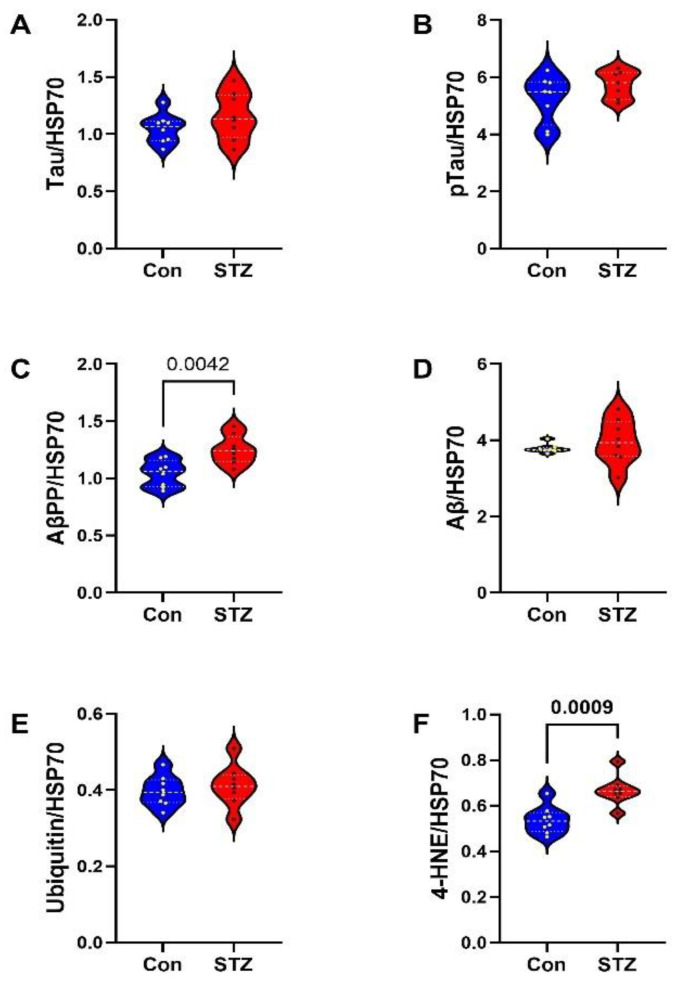
SEV AD and oxidative stress biomarkers: Violin plots compared SEV levels of (**A**) Tau, (**B**) pTau, (**C**) AβPP, and (**D**) Aβ1-42, (**E**) ubiquitin, and (**F**) 4-HNE immunoreactivities in control (Con) and i.c.-STZ (STZ) FLEVs isolated from Long Evans P30 rat brains (n = 8/group). Immunoreactivity was measured by duplex ELISA with results normalized to RPLPO. Intergroup comparisons were made by two-tailed *t*-tests with Bonferroni correction for repeated measures. Significant differences correspond to *p* < 0.05.

**Figure 10:**
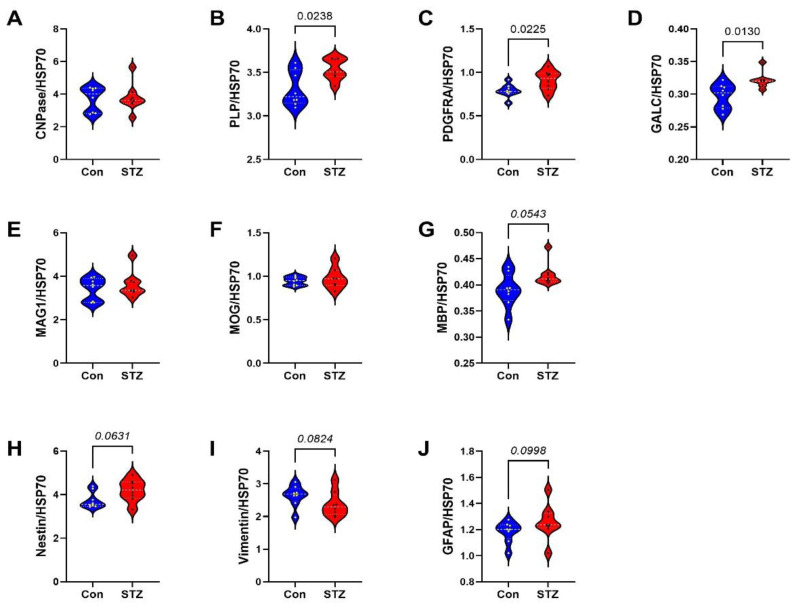
SEV glial protein expression: SEV samples from control (Con) and i.c. STZ (STZ) treated P30 Long Evans rats were used to measure (**A**) CNPase, (**B**) PLP, (**C**) PDGFRA, (**D**) GALC, (**E**) MAG, (**F**) MOG, (**G**) MBP, (**H**) Nestin, (**I**) vimentin, and (**J**) GFAP by duplex ELISA with results normalized to RPLPO. Assays were performed in triplicate with 8 samples/group. Intergroup comparisons were made by two-tailed *t*-tests with Bonferroni correction for repeated measures. Significant differences correspond to *p* < 0.05. Statistical trend-wise differences (italicized) reflect 0.05 < *p* < 0.10.

Duplex ELISAs detected significantly elevated levels of Aβpp (Figure 9C) and 4-HNE (Figure 9F) in the STZ SEV samples and no significant effects of STZ on Tau (Figure 9A), pTau (Figure 9B), Aβ (Figure 9D), or ubiquitin (Figure 8E). Analysis of SEV glial protein expression revealed significantly elevated levels of PLP (Figure 10B), PDGFRA (Figure 10C), and GALC (Figure 10D) and trend-wise increases in MBP (Figure 10G), Nestin (Figure 10H), vimentin (Figure 10I), and GFAP (Figure 10J) in the STZ samples. In contrast, no significant or trend-wise STZ effects were observed with respect to CNPase (Figure 10A), MAG (Figure 10E), or MOG (Figure 10F).

**Figure 11:**
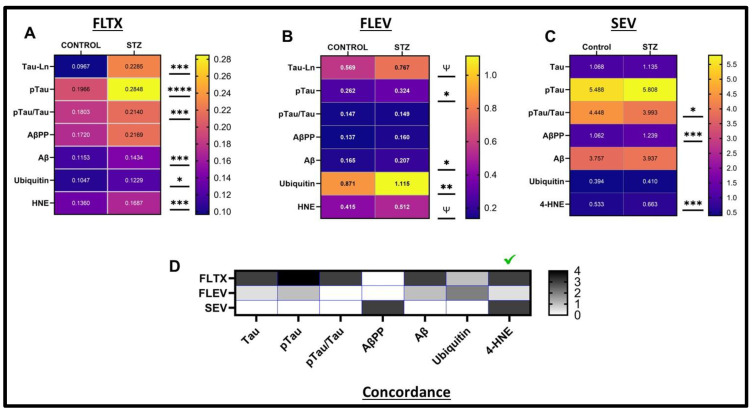
AD and oxidative stress biomarker expression heatmaps. Heatmap displays of STZ effects on AD biomarkers and oxidative stress molecule expression in (**A**) FLTX, (**B**) FLEV, and (**C**) SEV. The scale bars reflect graded differences in the levels of immunoreactivity as depicted in Figure 3, Figure 6, and Figure 9. Significant (* *p* < 0.05; ** *p* < 0.01; *** *p* < 0.001; **** *p* < 0.0001) and trend-wise statistical differences (ψ 0.05 < *p* < 0.10) are displayed adjacent to each heatmap. (**D**) Concordant responses to STZ in FLEV vs. FLTX and SEV vs. FLTX are displayed with a heatmap. Shading intensities correspond with the *p*-values (asterisks) for STZ effects shown in the Heatmaps.

Heatmap Comparisons of AD Biomarkers: The AD biomarker ELISA results were re-configured using heatmaps to compare the effects of STZ across the FLTX (Figure 11A), FLEV (Figure 11B), and SEV (Figure 11C) samples, and the results were analyzed by two-way ANOVA tests (Table 1). Due to the high levels of Tau in FLTX and FLEV, the values were Ln-transformed for comparisons with the other much less abundantly expressed biomarkers. In addition, data corresponding to the calculated pTau/Tau ratios are included in the heatmaps. To illustrate the concordant effects of STZ across sample types, a heatmap was generated depicting the significance levels (*p*-values) associated with the STZ effects (Figure 11D).

Side-by-side comparisons revealed STZ-associated increases in Tau, pTau, AβPP, Aβ, ubiquitin, and 4-HNE in the FLTX and FLEV samples; discordant significant increases in pTau/Tau in FLTX but not FLEV; and concordant absent STZ effects on AβPP. Therefore, most of the STZ effects on AD biomarkers in FLTX were also detected in FLEV. In contrast, FLTX-SEV concordant effects of STZ were limited and observed only for 4-HNE. The significant effect on pTau/Tau was directionally opposite (reduced in SEV and increased in FLTX), and AβPP was discordantly increased in SEV, with an effect on FLTX. Correspondingly, the concordance heatmap (Figure 11D) shows that only 4-HNE was increased in FLTX, FLEV, and SEV, whereas the main AD biomarkers were concordantly increased in FLTX and FLEV but not SEV.

**Figure 12:**
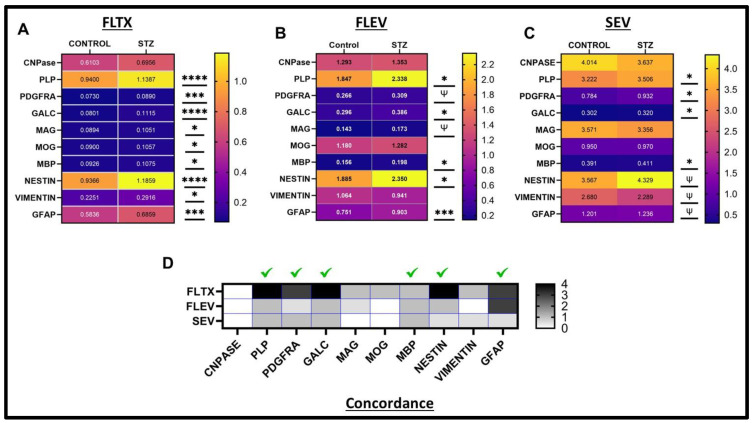
Glial protein expression heatmaps. Heatmap displays of STZ effects on glial molecule expression in (**A**) FLTX, (**B**) FLEV, and (**C**) SEV. The scale bars reflect graded differences in the levels of immunoreactivity as depicted in Figure 4 and Figure 7, and 10. Significant (* *p* < 0.05; *** *p* < 0.001; **** *p* < 0.0001) and trend-wise statistical differences (ψ 0.05 < *p* < 0.10) are displayed adjacent to each heatmap. (**D**) Concordant responses to STZ in FLEV vs. FLTX and SEV vs. FLTX are displayed with a heatmap. Shading intensities correspond with the *p*-values (asterisks) for STZ effects shown in the heatmaps.

Heatmap Comparisons of White Matter/Glial Biomarkers: The heatmap corresponding to glial marker data demonstrated uniformly low levels of PDGFRA, GALC, MAG, MOG, and MBP relative to CNPase, PLP, Nestin, and GFAP in both control and STZ FLTX but consistently higher levels of all except CNPase in STZ versus control FLTX samples (Figure 12A). Compared with FLTX, the FLEV samples exhibited remarkably similar profiles of glial protein immunoreactivity, with concordant STZ-associated increases in PLP, PDGFRA, GALC, MAG, MBP, Nestin, and GFAP (Figure 12A,B,D). The main differences were that MOG was expressed at approximately 10-fold higher levels in FLEVs compared with FLTX and 5- to 10-fold higher than PDGFRA, GALC, MAG, and MBP in the same FLEV samples. In addition, STZ increased vimentin in FLTX but had no significant or trend-wise effect in FLEVs. The SEV heatmap demonstrated striking differences in the relative abundance, i.e., primarily higher levels of the different glial markers compared with FLTX and FLEV (Figure 12C). In the SEVs, MAG expression was similar to CNPase and PLP, whereas in FTLX and FLEV, MAG expression was considerably lower than CNPase and PLP. Like FLTX and FLEV, PLP, PDGFRA, GALC, MBP, Nestin, and GFAP immunoreactivities were significantly or statistically trend-wise increased by STZ, whereas CNPase was unaffected. However, discordant SEV responses to STZ relative to FLTX or FLEV were marked by reduced vimentin and absent changes in MOG and MAG. The concordance heatmap (Figure 12D) illustrates that significant STZ-associated abnormalities in FLTX glial markers, including PLP, PDGFRA, GALC, MBP, Nestin, and GFAP, are detectable in both FLEVs and SEVs.

## 3. Discussion

AD diagnostics, monitoring, and treatment continue to be challenged due to difficulties in identifying all facets of the disease, particularly in its early stages. Genuine progress in AD therapeutics will require assurances that the full spectrum of neurodegeneration is addressed. Moreover, the monitoring of treatment interventions should become streamlined, rapid, cost-effective, and minimally invasive. Growth in the field of exosome research offers hope for achieving comprehensive evaluations of organ-system pathologies with minimally invasive methodologies. For example, exosome-based approaches have become widespread in cancer diagnostics [11,22,36]. However, such successes have been linked to narrow requirements of biomarker detection. With neurodegeneration and non-neoplastic diseases in general, there are no unique biomarkers because cellular, tissue, and organ pathophysiological changes are complex and evolve over time. However, the complexity of degenerative disease processes renders them ideally suited for multi-marker minimally invasive exosome-based diagnostics.

This study utilized an experimental model of i.c. STZ, which, in previous studies, was shown to cause neurodegeneration with pathologies reminiscent of sporadic AD in humans [56,57,58]. The i.c. STZ model features deficits in neurocognitive function, increased brain levels of Aβ, pTau, oxidative stress, white matter atrophy with oligodendrocyte dysfunction, impairments in insulin and IGF signaling mechanisms, and alterations in neuropeptide expression. The unique feature of this study is that biomarker expression profiles that mark AD-type neurodegeneration, oxidative stress, and changes in white matter integrity in the frontal lobe were simultaneously characterized in exosomes isolated from frontal tissue and serum. This strategy was utilized to gain a better understanding of the degree to which central nervous system neuropathological processes are reflected in exosomes circulating in peripheral blood, to better assess the utility and direct development of liquid biopsy approaches to diagnose and monitor neurodegeneration, including disease progression and responses to treatment.

Clinical tests routinely measure Aβ and pTau in serum and CSF to aid in diagnosing AD. Although those standard assays are performed without the need to isolate exosomes, the findings herein indicate that at least subsets of AβPP, Aβ, Tau, and pTau are distributed in exosomes. At this point, it is not clear if these molecules circulate only within exosomes or also as free molecules. This concept was confirmed by the finding of residual AβPP, Aβ, Tau, and pTau immunoreactivities in remnant serum samples after the exosomes had been isolated. Additional studies are needed to better understand the factors and conditions that lead to exosome-associated versus exosome-free AβPP, Aβ, Tau, and pTau. Nonetheless, regarding these biomarkers, as expected, higher levels of Tau, pTau and Aβ were measured in STZ FLTX relative to control, consistent with previous reports [2]. The higher STZ frontal lobe levels of ubiquitin and 4-HNE reflect additional evidence of neurodegeneration with accumulation of the misfolded proteins targeted for degradation via the ubiquitin–proteosome pathway [59] and increased oxidative stress with lipid peroxidation. Altogether, these findings in the STZ model correspond with neurodegeneration marked by brain atrophy and impaired performance on the Morris Water Maze test, consistent with previous reports [5]. It is also noted that, although the i.c. STZ causes insulin deficiency with cognitive impairment and neurodegeneration, over time, i.c. STZ-treated animals exhibit body weight gains, hepatic steatosis (fatty liver), and peripheral insulin resistance [5,60], which likely account for the statistical trend-wise increases in liver and body weight observed herein in the i.c. STZ group.

For the exosome/EV studies, we first demonstrated their characteristics in relation to treatment (STZ or vehicle) and source (FLTX or serum). There were several notable differences between the FLEVs and SEVs. FLEVs had broader size range profiles than SEVs, with peaks around 150 nm in FLEVs versus 100 nm in SEVs. In addition, small peaks corresponding to EVs larger than 400 nm were observed in FLEVs but not in SEVs. Consequently, the mean particle sizes were larger in FLEV compared with SEV samples. On the other hand, the nanoparticle concentration was lower in control FLEV versus control SEV samples. STZ treatment had significant effects on the particle concentrations, increasing in FLEV but reducing in SEV. In addition, STZ SEVs were significantly smaller than control SEVs. Importantly, these observations highlight how treatments and disease states can alter EV generation and size profiles, which may themselves serve as biomarkers of pathology.

An important observation enabling comparisons of EV immunoreactivity was that the mean levels of tetraspanin immunoreactivity and HSP70 normalized for tetraspanin immunoreactivity were similar for control and STZ samples. The CD9 + CD63 + CD81 cocktail of tetraspanins was used because it proved a more stable index relative to protein concentration than individual tetraspanins. Regarding the AD and oxidative stress markers, the STZ-associated increases in immunoreactivity were largely mirrored by statistically significant or trend-wise effects in the FLEVs. Therefore, the AD-related molecular abnormalities present in frontal lobe tissue were, for the most part, detectable in FLEVs. In contrast, the only marker with concordant STZ-associated increased immunoreactivity was 4-HNE, indicating that most of the AD-related biomarkers were not concordantly shifted with either FLTX or FLEV. On the other hand, AβPP immunoreactivity was significantly increased in STZ-SEVs but not STZ-FLTX or STZ-FLEV. These findings suggest that analysis of unselected SEVs analysis, i.e., including circulating EVs from all sources, may not be feasible for detecting altered brain tissue AD biomarker expression. This point is particularly evident based on the concordance heatmap shown in Figure 11, marking the dissimilar profiles of AD biomarker immunoreactivity in SEVs compared with FLTX and FLEV but mainly similar profiles in FLTX and FLEVs. Potential explanations for the poor representation of AD biomarkers in SEVs are as follows: (1) Aβ and pTau primarily distribute exosome-free in serum, perhaps due to their release by degenerating or dying brain cells; or (2) pathological shifts in Aβ and pTau may require targeted analysis of enriched, brain-specific EVs.

One of the research goals was to determine if non-standard indices of AD-type neurodegeneration were detectable in EVs. Our efforts focused on oligodendrocyte, myelin, and neuroglial molecules because white matter degeneration is an important feature of AD, as it begins early and progresses with disease severity [42,61,62]. The STZ model was suitable because previous studies demonstrated significant alterations in white matter glial proteins and mRNA transcripts associated with brain atrophy [56]. The working hypothesis was that exosomes released due to white matter degeneration and/or attendant repair/recovery/remodeling traffic from the CNS into the peripheral circulation and, therefore, may be detectable in EVs. To this end, the markers evaluated included immature oligodendroglial (CNPase and PLP), non-myelinating glial (PDGFRA and GALC), mature oligodendrocyte (MAG, MOG, MBP), and astrocyte (Nestin, vimentin, GFAP).

Three of the four immature or non-myelinating glial proteins were increased in both FLTX and FLEV samples from STZ-treated rats. CNPase was the only immature marker unaffected by STZ. Of note is that white matter injury is known to be associated with the de-differentiation of oligodendrocytes coupled with altered expression of myelin/oligodendrocyte glycoproteins [44]. Conceivably, the STZ-associated increases in PLP, PDGFRA, and GALC mark oligodendrocytes’ injury and de-differentiation-related responses to white matter atrophy and degeneration. Importantly, concordant STZ-related increases in PLP, PDGFRA, and GALC were also detected in SEVs, indicating that indices of brain white matter molecular abnormalities were detected in SEVs isolated from serum.

Among the mature myelin/oligodendrocyte proteins, significant STZ-associated increases in MAG, MOG, and MBP were observed in FLTX, whereas corresponding trend-wise or significant increases in MAG and MBP but not MOG were observed in FLEV, and in SEV, only MBP was trend-wise elevated. Therefore, the only concordant STZ-related change detected in FLTX, FLEV, and SEV was increased MAG immunoreactivity. Since MAG inhibits neuritic outgrowth in myelinated axons [63,64], its elevated levels in EVs could reflect increased budding and exosome incorporation of damaged or degenerated mature oligodendrocyte/myelin membranes [63,64].

Nestin and vimentin are multilineage markers expressed in immature or progenitor cells, including glia [65]. Vimentin and GFAP are intermediate filament proteins that support the glial cytoskeleton and cell shape. Nestin, vimentin, and GFAP were all significantly increased in FLTX by i.c. STZ, whereas in FLEVs, only nestin and GFAP were significantly elevated. In SEVs, the effects of STZ were all trend-wise, increased for nestin and GFAP but reduced for vimentin. Therefore, STZ-related alterations in FLTX astrocyte/glial markers were concordantly increased for nestin and GFAP in FLEVs, but in the SEVs, they were only marginally elevated with respect to nestin and GFAP but discordantly reduced for vimentin. The increased levels of nestin and GFAP in FLTX and EVs may reflect astrocyte responses to white matter degeneration. However, as noted for the AD biomarker, STZ-mediated alterations in FLTX expression of glial/astrocyte markers were not convincingly detected and not uniformly concordant. Therefore, with unselected SEVs, the detection of STZ/AD-associated glial-astrocytic brain pathologies would be challenging.

## 4. Materials and Methods

Materials: Streptozotocin (STZ) was purchased from Millipore-Sigma (Burlington, MA USA). Epitope-specific commercial primary antibodies used in duplex enzyme-linked immunosorbent assays (ELISAs), including their sources, concentrations used, vendors, and catalog or Research Resource Identifier (RRID) numbers, are listed in Table 2. Bicinchoninic acid (BCA) reagents, ELISA MaxiSorp 96-well plates, horseradish peroxidase (HRP)-conjugated secondary antibodies, and superblock (TBS) were obtained from ThermoFisher Scientific (Bedford, MA, USA). The Amplex ultrared soluble fluorophore was acquired from Life Technologies (Carlsbad, CA, USA). All other fine reagents were obtained from Sigma-Aldrich Co. (St. Louis, MO, USA), CalBiochem/Millipore Sigma (Burlington, MA, USA), and Pierce Chemical (Dallas, TX, USA). The Total Exosome Isolation Kit reagents were from Invitrogen/Life Technologies.

Experimental Model: Long Evans postnatal day 3 (P3) rats (Charles River Laboratories, Willmington, MA, USA) were rendered hypothermic prior to administering 25 mg/kg i.c. STZ into the frontal horns of the lateral ventricles [57,66,67]. The control group was treated with i.c. saline. The STZ and control groups were each generated with 16 rat pups (8 males and 8 females) drawn from 6 litters. The rats were maintained in a pathogen-free environment with unrestricted access to food, air temperature between 23 °C and 25 °C, and automated 12 h light/dark cycles (7AM lights on−7PM lights off). The use of rats for these experiments was approved by the Institutional Animal Care and Use Committees (IACUC) at Lifespan (Committee #2005450 and #1571553), and the protocols adhered to the Care and Use of Laboratory Animals publication from the National Institutes of Health.

Morris Water Maze: The Morris Water Maze (MWM) test of spatial learning and memory was performed over 4 consecutive days from P24 to P27, as previously described [57]. In brief, on Trial Day 1, the rats were oriented to the MWM and taught how to locate and land on the platform in the center of the maze. On the subsequent 3 days, the rats were progressively challenged by modifying the entry points and submerging the platform. For each trial, the rats swam for up to 120 s to independently locate and land on the platform, after which they were guided to the platform. Each of the 3 daily trials was separated by 5 to 10 min of rest. After each trial, the rats were towel-dried and kept warm for comfort. EthoVision 8.5 (Noldus, Leesburg, VA, USA) was used to video-record path length, path complexity, velocity, latency, and errors. However, the data analysis was focused on latency due to similar swimming speeds in the control and STZ-treated rats. Longer latencies were associated with more errors and increased path complexity and length. Inter-group comparisons were based on area-under-the-curve calculations for latency over the 3 trials in each rat. Area under the curve provides cumulative integrated measurements of treatment effects.

Sample Harvesting: At the P31 or P32 experimental endpoint, the rats were sacrificed by isoflurane inhalation. Non-hemolyzed serum samples obtained by cardiac punction were aliquoted, stored, and frozen at −80 °C. After removing the brain, the frontal lobes were harvested by making a pre-temporal tip coronal slice, and the samples were divided, snap-frozen, and stored for later molecular and biochemical studies. The molecular and biochemical assays were performed using 8 samples (4 males and 4 females)/group, which previous studies showed provides sufficient statistical power.

Frontal Lobe Homogenates: Using a TissueLyser II (Qiagen, Germantown, MD, USA) and 5 mm diameter stainless-steel beads, individual fresh frozen FL tissue samples (50 mg each) were homogenized in 5 volumes of weak lysis buffer (50 mM Tris (pH 7.5), 150 mM NaCl, 5 mM EDTA (pH 8.0), 0.1% Triton X-100) supplemented with protease (1 mM PMSF, 0.1 mM TPCK, 2 µg/mL aprotinin, 2 µg/mL pepstatin A, 1 µg/mL leupeptin) and phosphatase (10 mM Na3VO4) inhibitors. The supernatants obtained by centrifuging the samples at 14,000 rpm for 10 min at 4 °C were aliquoted and stored at −80 °C for subsequent immunoassays.

Frontal Lobe Exosome (FLEV) Isolation: Brain tissue EVs were isolated as previously described [68]. In brief, fresh frozen FL tissue samples (25 mg) were lightly thawed for dounce homogenization in phosphate-buffered saline (PBS) containing protease and phosphatase inhibitors. Supernatants obtained by centrifuging the suspensions at 500× *g* for 5 min at 4 °C were transferred to fresh tubes and re-centrifuged at 2000× *g* for 10 min at 4 °C. FLEVs were isolated using the Total Exosome Isolation kit (Invitrogen, Carlsbad, CA, USA), following the manufacturer’s protocol. In brief, after adding the exosome isolation reagent and incubating for 30 min at 4 °C, the samples were centrifuged at 10,000× *g* for 10 min (RT), after which the supernatants were thoroughly removed without disturbing the pellets. The pellets were resuspended in PBS containing. Aliquots stored with 1% DMSO at −20 °C were used for NanoSite analysis. The remainder was resuspended in weak lysis buffer and stored at −80 °C.

Serum Extracellular Vesicle (SEV) Isolation: SEVs were isolated from serum using the Total Exosome Isolation reagents (Invitrogen/Life Technologies) following the manufacturer’s protocol. In brief, the frozen serum samples were thawed in a 25 °C water bath and then centrifuged at 2000× *g* for 30 min to remove debris. A 100 µL aliquot of clarified serum was added to 20 µL total exosome isolation reagent, vortex mixed to achieve a homogenous solution, incubated at 4 °C for 30 min, then centrifuged at 10,000× *g* for 10 min at room temperature. The pelleted exosomes were resuspended in 25 µL phosphate-buffered saline (PBS). Exosome isolation was confirmed by nanoparticle tracking analysis and ELISAs to measure tetraspanin immunoreactivity.

Nanoparticle Tracking Analysis (NTA): NTA was performed with a NanoSight NS500 instrument (Malvern Instruments, Malvern, UK) equipped with a syringe pump and NTA software v3.44 to determine the size distributions and number of particles per sample. For the NTA studies, DMSO was added to a final concentration of 1% for stabilization. The samples were analyzed in triplicate. Prior to sample analysis, the instrument was calibrated using Nanosphere Standard Beads (Thermo Scientific, Waltham, MA, USA). Subsequent continuous monitoring ensured the maintenance of optimized settings. Video recordings were used to evaluate the mean, median, and mode of the particle sizes and estimated concentrations.

Enzyme-Linked Immunosorbent Assay (ELISA) Studies: ELISAs were used to measure immunoreactivity to markers of AD neurodegeneration, oxidative stress, and glial proteins in FLTX, FLEV, and SEV samples. Tetraspanin immunoreactivity, measured with a CD9 + CD63 + CD81 antibody cocktail, and large acidic ribonuclear protein (RPLPO) [68,69,70,71,72] and heat shock protein 70 (HSP70) immunoreactivities served as additional positive controls. See Table 3 for the list of molecules assayed and their functions. The samples were homogenized in weak lysis buffer that contained protease and phosphatase inhibitors (see above). Protein concentrations were measured with the BCA assay. Sample aliquots containing 50 ng protein diluted in bicarbonate buffer were adsorbed to the bottom surfaces of 96-well ELISA MaxiSorp plates. Nonspecific sites were masked with Superblock-TBS. Triplicate sample wells were incubated overnight at 4 °C with primary antibodies. Immunoreactivity was detected with horseradish peroxidase-conjugated secondary antibody and the Amplex UltraRed soluble fluorophore. Fluorescence intensity (Ex530/Em590) was measured in a SpectraMax M5 microplate reader (Molecular Dynamics, Inc., Sunnyvale, CA, USA). The calculated ratios of target protein to large acidic ribonuclear protein (RPLPO) (for FLTX and FLEV) or HSP70 (for SEV) fluorescence were used for intergroup statistical comparisons.

Western Blot Analysis of EVs: FLEVs and SEVs used in ELISA studies were also subjected to Western blot analysis, as previously described [27,41]. In brief, EVs containing 8 µg protein were fractionated in 10% SDS-PAGE gels along with pre-stained molecular weight standards (Precision Plus Protein Dual Color Standards, Bio-Rad Laboratories, Hercules, CA, USA; Catalog #161-0374). The samples were electroblot transferred to polyvinylidene difluoride (PVDF) membranes (Bio-Rad Laboratories, Hercules, CA, USA) and incubated with SuperBlock-TBS (Thermo Fisher, Bedford, MA, USA) to mask nonspecific binding sites. The membranes were then incubated separately with primary antibodies to CD9, CD63, and CD81 overnight at 4 °C with gentle platform agitation. Immunoreactivity was detected with horseradish peroxidase (HRP)-conjugated secondary antibody and Femto-chemiluminescence reagent (Thermo Fisher Scientific; Prod #34096). Immunoreactivity was imaged using the Intelligent Dark Box Ⅱ (FUJIFILM, Tokyo, Japan).

Statistical Analyses: The data were analyzed using analysis of variance and two-tailed *t*-tests with corrections for multiple comparisons. Violin plots depict the median (mid-horizontal bar), the first (lower horizontal line) and third (upper horizontal line) quartiles, and range (tips) corresponding to the calculated relative levels of target molecule immunoreactivity. Heatmaps were used to compare patterns and levels of immunoreactivity in FLTX, FLEV, and SEV samples. Data were analyzed using repeated measures two-tailed *t*-tests with multiple comparison (Bonferroni) corrections. Two-way ANOVA tests compared heatmap results. Statistical analyses were performed with GraphPad Prism 10.4 software (La Jolla, CA, USA). Significant differences correspond to *p* < 0.05. Statistical trend-wise differences (italicized) reflect 0.05 < *p* < 0.10.

## 5. Conclusions

This study was designed to examine the degree to which molecular alterations in brains with neurodegeneration can be detected in serum EVs. The i.c. STZ model was used because it reproducibly causes cognitive impairment with deficits in spatial learning and memory, and many pathological features are reminiscent of sporadic AD in humans. A unique design of this experiment was that STZ-related shifts in the expression of AD biomarkers and indices of oxidative stress and oligodendrocyte/glial pathology were compared in FLTX, FLEVs, and SEVs. The main advantage over most other studies is that direct comparisons could be made across the different sample types, and the findings in SEV could be interpreted in light of known abnormalities in the brain. Importantly, the results demonstrated that a subset of oligodendrocyte/glial molecules rendered abnormal in FLTX were concordantly detected in SEVs, providing new opportunities to detect and monitor white matter degeneration using a non-invasive, EV-based liquid biopsy approach. The strengths of this study rest in its design, methodology, use of a well-studied reproducible model, and feasibility of the approach. The limitations include the relatively small sample sizes (n = 8/group), insufficient power to assess gender effects, and the analysis of total, unselected SEVs. Importantly, coupled with the current ATN classification system, which is based on Amyloid (Aβ42, Aβ42/Aβ40), Tau (p-Tau181, p-Tau217), and neurodegeneration (t-Tau, NfL), and neuroimaging to assess WM atrophy and other pathologies, exosome biomarkers of WM degeneration could help to further assess the risk of cognitive decline in relation to disease staging [4,73]. Future studies should focus on refining EV-related investigations via longitudinal evaluations that characterize shifts in the brain- and cell-type-specific SEV biomarker expression that reflect the full spectrum of AD neurodegeneration. The expectation is that these approaches will improve diagnostic accuracy and better delineate the subtypes of neurodegeneration that could be differentially targeted with treatments that address specific neuropathological processes. Finally, it may be possible to use the data to generate automated algorithms for early diagnosis and more accurate staging and prognostication of AD neurodegeneration.

## Figures and Tables

**Table 1:** Two-way ANOVA test results: heatmaps.

Variable	Treatment Effect-			Molecule Effect			Treatment × Molecule Effect		
FLTX	F-Ratio	DFn, DFd	*p*-Value	F-Ratio	DFn, DFd	*p*-Value	F-Ratio	DFn, DFd	*p*-Value
AD Biomarkers	16.89	1, 14	0.001	29.61	6, 84	<0.0001	6.795	6, 84	<0.0001
Glial Proteins	50.64	1, 140	<0.0001	754.0	9, 140	<0.0001	5.55	9, 140	<0.0001
FLEV	F-Ratio	DFn, DFd	*p*-Value	F-Ratio	DFn, DFd	*p*-Value	F-Ratio	DFn, DFd	*p*-Value
AD Biomarkers	24.17	1, 98	<0.0001	153.4	6, 98	<0.0001	3.176	6, 98	0.0069
Glial Proteins	26.35	1, 168	<0.0001	195.4	11, 168	<0.0001	3.291	11, 168	0.0004
SEV	F-Ratio	DFn, DFd	*p*-Value	F-Ratio	DFn, DFd	*p*-Value	F-Ratio	DFn, DFd	*p*-Value
AD Biomarkers	1.476	1, 96	N.S.	490.6	6, 96	<0.0001	2.978	6, 96	0.01
Glial Proteins	244.5	1, 136	<0.0001	*2.931*	9, 136	*0.089*	1.176	9, 136	N.S.

The ELISA results included in Figure 3, Figure 4, Figure 6, Figure 7, Figure 9 and Figure 10 were used to generate heatmaps and compare relative levels of AD biomarker and glial protein immunoreactivities in frontal lobe tissue (FLTX), frontal lobe tissue EVs (FLEV), and serum EVs (SEV). The results were analyzed by two-way ANOVA to demonstrate treatment, molecule, and treatment × molecule interactive effects on AD biomarker and glial protein expression. The F-ratios, degrees of freedom (DFn, DFd), and *p*-values are listed. Significant differences correspond to *p* < 0.05. Statistical trend-wise differences (italicized) reflect 0.05 < *p* < 0.10. See Heatmaps in Figure 11 and Figure 12.

**Table 2:** Commercial antibodies and sources.

Antibody Targets	Source	Monoclonal/ Polyclonal	Stock (mg/mL)	µg/mL or Dilution	Commercial Source	RRID or Reference *
CD9 (Tetraspanin-29)	Rabbit	Polyclonal	8.66	0.5	ABclonal, Woburn, MA, USA	A1703
CD63 (Tetraspanin-30)	Rabbit	Polyclonal	1.03	0.5	ABclonal, Woburn, MA, USA	A5271
CD81 (Tetraspanin-28)	Rabbit	Polyclonal	1.76	0.5	ABclonal, Woburn, MA, USA	A5270
HSP70 (Heat Shock Protein 70)	Rabbit	Polyclonal	1.84	0.5	ABclonal, Woburn, MA, USA	A0284
AβPP (Amyloid β-Precursor Protein)	Rabbit	Polyclonal	0.197	0.0985	Cell Signaling, Danvers MA, USA	Cat. #2452
Aβ (Amyloid β Peptide, 1-42)	Mouse	Monoclonal	0.2	0.8	Santa Cruz Biotechnology	Cat. #sc-28365
Tau (Tubulin-associated unit)	Rabbit	Polyclonal	6.2	3.1	Agilent/Dako, Santa Clara, CA, USA	REF A0024
pTau (PHF; S396) (Phosphorylated Tau	Mouse	Monoclonal	1.21	0.40	Cell Signaling, Danvers MA, USA	#9632
HNE (4-hydroxy-2-nonenal)	Goat	Polyclonal	0.8	1.6	Abcam, Boston, MA, USA	ab46544
Ubiquitin	Rabbit	Polyclonal	0.25	0.5	Abcam, Boston, MA, USA	ab7780-500
CNPase (11-5B) (2′,3′-cyclic nucleotide 3′ phosphodiesterase)	Mouse	Monoclonal	1.0	2.0	Abcam, Boston, MA, USA	ab6319
GALC (Group-specific component Vitamin D Binding protein; Gc-globulin)	Rabbit	Polyclonal	1.0	2.0	Abcam, Boston, MA, USA	ab83752
MAG (Myelin-Associated Glycoprotein 1)	Mouse	Monoclonal	0.5	0.25	Abcam, Boston, MA, USA	ab89780
MOG (Myelin Oligodendrocyte Glycoprotein)	Rabbit	Polyclonal	1.0	2.0	Abcam, Boston, MA, USA	ab32760
MBP (Myelin basic protein)	Rabbit	Polyclonal	1.0	2.0	MilliporeSigma, Burlington, MA, USA	M3821
PLP (Proteolipid Protein 1)	Rabbit	Polyclonal	Serum	1:2000	Abcam, Boston, MA, USA	ab28486
PDGFRA (Platelet-derived growth factor receptor, alpha polypeptide)	Rabbit	Polyclonal	1.0	1.0	Abcam, Boston, MA, USA	ab61219
Nestin	Rabbit	Polyclonal	Serum	1:2000	Abcam, Boston, MA, USA	ab27952
Vimentin	Mouse	Monoclonal	1.0	2.5	Abcam, Boston, MA, USA	ab8978
GFAP (Glial Fibrillary Acidic Protein)	Goat	Polyclonal	0.5	0.5	Abcam, Boston, MA, USA	ab53554
RPLPO (Large acidic ribosomal protein)	Mouse	Monoclonal	0.1	0.1	Proteintech, Chicago, IL, USA	AG1829

* RRID = Research Resource Identifier; references refer to literature citations with validations of RPLPO antibody.

**Table 3:** Tetraspanin and neuroglial proteins and their functions.

Abbreviation	Full Name	Gene Names	Product Functions
CD9	Tetraspanin-29	*CD9*, *TSPAN29*	Many cellular processes including adhesion, differentiation, signal transduction, suppression of cancer cell motility, and metastatic spread.
CD63	Tetraspanin-30	*CD63*, *TSPAN30*	Cell surface glycoprotein that complexes with integrins. CD63 is associated with tumor progression.
CD81	Tetraspanin-28	*CD81*; *TSPAN28*	Cell surface glycoprotein that complexes with integrins, promotes muscle cell fusion, supports myotube maintenance, has roles in signal transduction and possibly tumor suppression in malignancies.
HSP70	Heat Shock Protein 70	*HSPA*	A critical 70 kDa molecular component of cellular machinery utilized for protein folding and protecting cells from stress.
CNPase	2′,3′-cyclic nucleotide 3′ phosphodiesterase	*CNP*, *EC*, *CNP1*	Myelin-associated marker of oligodendrocytes and Schwann cells that may play an important role in the development of myelin membranes and sustain axonal integrity.
PLP1	Proteolipid Protein 1	*PLP*; *SPG2*	Transmembrane proteolipid protein, dominant in CNS myelin. It may be involved in compaction, stabilization, and maintenance of myelin sheaths, oligodendrocyte development, and axonal survival.
PDGFRA	Platelet Derived Growth Factor Receptor, alpha polypeptide	*PDGFRA*; *CD140a*	Cell surface tyrosine protein kinase receptor required for skeleton development and cephalic closure during embryonic development. Survival factor for oligodendrocyte progenitor cells.
GALC	Galactosylceramidase	*GALC*; *Galactosylceramidase*	Encodes a lysosomal protein that catabolizes/ hydrolyzes galactose ester bonds of galactosylceramide, lactosylceramide and galactosylsphingosine; major lipid in myelin.
MAG	Myelin Associated Glycoprotein	*MAG*; *GMA*	Glycoprotein that facilitates sialic acid-dependent cell-cell interactions between neuronal and myelinating cells. Found on oligodendrocytes and Schwann cells.
MOG	Myelin Oligodendrocyte Glycoprotein	*MOGIG2*	Expressed on oligodendrocyte cell surfaces and the outer surface of myelin sheaths. It may be involved in the completion or maintenance of myelin sheaths.
MBP	Myelin Basic Protein	*MBP*; *Myelin A1 Protein*	Major component of myelin sheaths in both oligodendrocytes and Schwann cells. Aids in the formation and stabilization of myelin membranes.
NES	Nestin	*NES*	Intermediate protein promoting disassembly of phosphorylated vimentin during mitosis. Required for survival, renewal, and mitogen-stimulated proliferation of neural progenitor cells.
VIM	Vimentin	*VIM*; *CTRCT30*	Class-III intermediate filament that maintains cell shape and cytoplasm integrity, stabilizing cytoskeletal interactions. May be involved in peripheral nerve myelination.
GFAP	Glial Fibrillary Acidic Protein	*GFAP*; *Intermediate Filament Protein*	Astrocyte intermediate filament cytoskeletal protein.

## Data Availability

The data underlying this article will be shared upon reasonable request to the corresponding author.

## Supplementary Materials

The following supporting information can be downloaded at: https://www.mdpi.com/article/10.3390/ijms26094190/s1.

## Abbreviations

**Abbreviation**	**Definition**
4-HNE	4-hydroxy-2-nonenal
AD	Alzheimer’s Disease
Aβ	Amyloid β Peptide, 1-42)
AβPP	Amyloid β-Precursor Protein
BCA	Bicinchoninic Assay
CNPase (11-5B)	2′,3′-cyclic nucleotide 3′ phosphodiesterase
CSF	Cerebrospinal Fluid
ELISA	Enzyme-Linked Immunosorbent Assay
EV	Extracellular Vesicles or Exosomes
FLEV	Frontal Lobe Extracellular Vesicles
FLTX	Frontal Lobe Tissue
GALC	Group-specific component Vitamin D Binding protein; Gc-globulin
GFAP	Glial fibrillary acidic protein
HRP	Horseradish Peroxidase
HSP70	Heat Shock Protein 70
MAG	Myelin-Associated Glycoprotein
MBP	Myelin basic protein
MOG	Myelin Oligodendrocyte Glycoprotein
MWM	Morris water maze
NTA	Nanoparticle Tracking Analysis
PDGFRA	Platelet-derived growth factor receptor, alpha polypeptide
PLP	Proteolipid Protein 1
pTau (PHF; S396)	Phosphorylated Tau
RPLPO	Large acidic ribonuclear protein
SEV	Serum Extracellular Vesicles
STZ	Streptozotocin
Tau	Tubulin-associated unit
WM	White Matter
