# Supplementary figures and images for: Comparative Brain and Serum Exosome Expression of Biomarkers in an Experimental Model of Alzheimer-Type Neurodegeneration: Potential Relevance to Liquid Biopsy Diagnostics
Source: Int J Mol Sci. 2025 Apr 28;26(9):4190. doi: 10.3390/ijms26094190 (PMC12071450; doi:10.3390/ijms26094190)

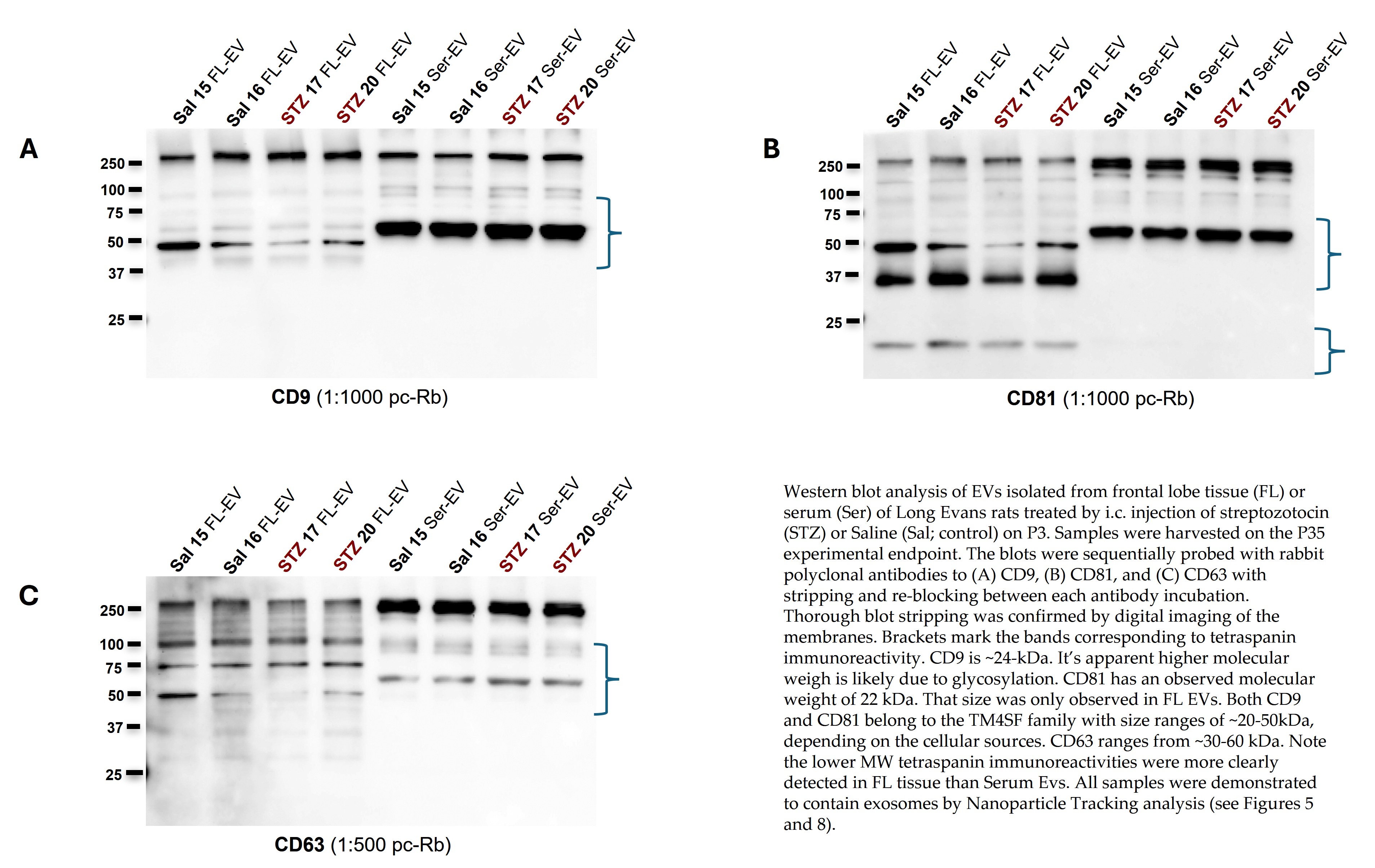

Supplement: Supplementary file 1 [file ijms-26-04190-s001.zip › ijms-3549208-supplementary.jpg]
